# Wasting syndrome as a predictive factor for adverse health outcomes in older adults: A prospective cohort study

**DOI:** 10.1002/ncp.70033

**Published:** 2025-09-23

**Authors:** Roana Carolina Bezerra dos Santos, Cláudia Porto Sabino Pinho, Eduila Maria Couto Santos, Maria Caroline da Silva Patricio, Stefany Beatriz do Nascimento, Letícia Sabino Santos, Taynara de Sousa Rego Mendes, Alcides da Silva Diniz

**Affiliations:** ^1^ Department of Nutrition Federal University of Pernambuco Recife Pernambuco Brazil; ^2^ Hospital of Clinics Federal University of Pernambuco Recife Pernambuco Brazil; ^3^ Brazilian Company of Hospital Services (EBSERH) Recife Pernambuco Brazil

**Keywords:** mortality, older adults, prognosis, survival, weight loss

## Abstract

Wasting syndrome (WS), also known as unintentional weight loss, is defined as a 5% reduction in body weight over a period of 6–12 months. The mortality associated with WS in older adults has not been adequately explored. The objective of this study was to investigate this relationship in hospitalized older individuals over an 18‐month period. 175 older adults who were hospitalized were observed for 18 months to observe outcomes such as new hospitalizations within this timeframe, length of hospital stay, and death. Weight loss was defined as the difference between measured weight and self‐reported weight over the last 6–12 months. Outcomes were obtained through telephone tracking. WS was observed in 45.7% of older individuals at study baseline, with 46.8% experiencing hospital stays of ≥10 days. 26.9% of these patients died, and 43.4% were rehospitalized during the follow‐up. Deaths occurred earlier among patients with WS compared with unexposed individuals (*P* = 0.028) according to the Kaplan‐Meier curve, but in the Cox regression WS was not significantly associated with mortality. In conclusion, a high prevalence of WS was observed in hospitalized older people, with a significant association with functional dependence. Although WS was not an independent predictor of mortality in the multivariate model, it was associated with earlier deaths in the unadjusted survival analysis.

## Graphical abstract

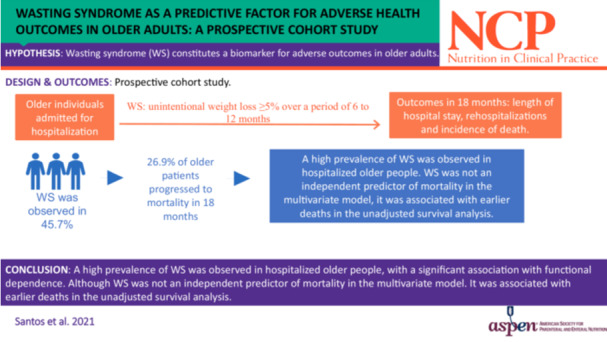

## INTRODUCTION

Wasting syndrome (WS), also known as unintentional weight loss, is defined as a 5% reduction in body weight over a period of 6–12 months, without a known cause, with prevalence rates that can reach 15%–20% in older adults.^1^ WS in patients aged >65 years is related to functional decline, increased hospitalization rates, and cachexia.^2^

Nutrition assessment strategies, such as the Global Leadership Initiative in Malnutrition (GLIM) criteria, incorporate weight loss among the phenotypic criteria for diagnosing malnutrition, recognizing the importance of this variable as a health biomarker. However, the evaluation of the GLIM criteria includes the analysis of other parameters, which are not always available, such as inflammation, muscle mass reduction, food intake, and body mass index. Involuntary weight loss can precede malnutrition, but it is often a neglected sign when the sum of the criteria does not meet the required number of criteria to establish the diagnosis of malnutrition.^3^

The mortality associated with WS in older adults has not been adequately explored. Studies in specific populations have revealed variation between 16% and 38%, especially in patients diagnosed with malignant diseases.^4^, ^5^ The duration of hospitalizations for older individuals with WS is a relevant concern for healthcare services, as these patients often face prolonged hospital stays because of the complexity of diagnosis and treatment and the need for a multidisciplinary approach.^6^, ^7^ Additionally, hospital readmissions can be a common complication in individuals with WS, imposing an additional burden on the healthcare system and impacting the quality of life of affected patients.^7^, ^8^ Therefore, we hypothesize that WS constitutes a biomarker for adverse outcomes in older adults.

WS constitutes an easily obtainable measure by the multidisciplinary healthcare team. Recognizing its predictive role in unfavorable events can reinforce the importance of including this parameter in clinical anamneses, favoring the adoption of management and intervention measures, and highlighting the importance of monitoring the course of the weight curve and significant and involuntary weight loss in older adults. Few studies so far have focused on the assessment of isolated weight loss.

Although there are some studies addressing the issue, these are often limited by small samples, a cross‐sectional design, and variable populations, and there are few specific recent studies involving older patients.^2^, ^8^ In this context, this study aimed to investigate the predictive capacity of WS to predict adverse outcomes in older adults over an 18‐month period after hospitalization.

## METHODS

This is a prospective cohort study involving older individuals of both sexes aged ≥60 years who were hospitalized in the clinical and surgical wards at the Clinics Hospital of Federal University of Pernambuco, Brazil. The study considered assessments of older individuals admitted for hospitalization from May to November 2021. The initial data collection (baseline–T0) involved gathering nutrition and clinical information. Patients were followed for 18 months (with a margin of another 4 months) to observe outcomes (T1): length of hospital stay, rehospitalizations in the reference service, and incidence of death.

The hospital service setting of this investigation represents a public, tertiary, university hospital located in an urban area in northeast Brazil, which provides multiple clinical and surgical specialties: cardiovascular disease, vascular, neoplasms, renal disease, gynecological and urological, psychiatric, orthopedic, pulmonary, endocrine, rheumatological, infectious diseases, and digestive system diseases.

Patients with a decreased level of consciousness; edematous and/or with ascites; with physical disabilities such as amputations, paraplegia, tetraplegia, or hemiparesis; and those for whom weight loss could not be documented were excluded. These criteria were considered because they hindered the direct and standardized measurement of body weight.

The sample size was estimated using the Statcalc program of the EPI‐INFO software (version 6.04; World Health Organization [WHO]/Centers for Disease Control and Prevention [CDC]) based on the following parameters: significance level of 95% (1−*α*); a study power of 80% (1−*β*); a ratio of 2:1, considering exposure (WS); and a relative risk for mortality equal to 2.0.^9^ For a finite population, a minimum sample size of 173 participants was found. To account for potential losses during the study, an additional 10% was added, resulting in a total sample of 191 individuals.

### Study operation and data collection

At baseline (T0), patient data were collected during the first 72 h after admission. In the assessment of outcomes (T1), 18 months after baseline, patients were tracked via phone contact, with information on death, survival, and rehospitalizations obtained from the patients or their families. Medical records were also examined to verify the occurrence of readmissions during the investigated period.

All assessments at the study baseline and phone interviews were conducted by trained clinical nutritionists following the study protocol to ensure standardized data collection. Under no circumstances did the researcher intervene or express personal opinions that could influence the response or interfere with the research outcome.

### Assessment of WS

WS was determined based on unintentional weight loss ≥5% over a period of 6–12 months, considering the following formula: weight loss = ([usual weight in 6–12 months−current weight] × 100/usual weight).^1^ To obtain the current weight, patients were weighed in an upright position, wearing light clothes and barefoot, using a digital anthropometric medical scale (Welmy W200A), with a maximum capacity of 200 kg and a variation of 100 g. The usual weight was obtained from the patient's report.

### Clinical outcomes

Unfavorable clinical outcomes during the 18‐month monitoring included new hospitalization in this timeframe, length of hospital stay, and death. Hospital stay duration was considered prolonged if it was ≥10 days according to the median of the population.^10^

### Sociodemographic, clinical, and functional variables

Regarding sociodemographic variables, data related to sex (male and female) and age (categorized into 60–69 years and ≥70 years) were collected.

For clinical variables, the presence of comorbidities such as systemic arterial hypertension (SAH), diabetes mellitus (DM), and clinical diagnoses were assessed from the medical record in the patient medical record. Clinical diagnoses were categorized into two groups: (1) malignancies (all types of cancers) and (2) nonmalignant organic disorders (digestive disorders, endocrine disorders, infectious diseases, nervous system diseases, respiratory diseases, systemic autoimmune diseases, and kidney and ureteral diseases).^2^, ^11^ The primary clinical diagnosis that required hospitalization was considered for the purpose of analysis.

Functional dependence was assessed through the Barthel Index, which evaluates activities of daily living (ADLs). Those who scored the maximum points (100 points) were considered totally independent, mild dependence was 99–76 points, moderate dependence was 75–51 points, severe dependence was 50–26 points, and total dependence was ≤25 points.^12^ For analysis purposes, the classification of dependence was dichotomized into functional independence (independence, score equal to 100 for the index) and functional dependence (dependence, score ≤100).

### Statistical analysis

The collected data were analyzed using the statistical software SPSS (version 20.0; SPSS Inc.). All continuous variables were tested for symmetry, and those with a symmetric distribution were described as mean and SD, whereas those with a nonsymmetric distribution were described as median and interquartile range (IQR). Categorical variables were expressed as proportions and corresponding percentages.

Univariate analysis was performed between WS and baseline measured covariates, as well as WS and outcomes, using Pearson chi‐square test, estimating prevalence ratios (PRs) and their respective 95% CIs.

Survival curves with potential predictors of mortality were estimated using the Kaplan‐Meier method. The corresponding *P* value was calculated using the log‐rank test (Mantel‐Cox). The estimation of the effect of covariates was performed by the semiparametric proportional hazards model, the Cox model, which estimates the proportionality of risks throughout the entire observation time. The assumption of proportional hazards for the Cox model was verified by analyzing Schoenfeld residuals. The overall fit quality of the model to the data was checked through Cox‐Snell residuals analysis. To assess the relationship between potential predictors of mortality (primary outcome), hazard ratios (HRs) with respective CIs and the Wald test were employed through Cox regression models, both in crude form (isolated variables) and after adjustment (model with all variables).

### Ethical aspects

The research was approved by the Research Ethics Committee of the Clinics Hospital under CAAE: 57869122.3.0000.8807 following resolution no. 466/2012 of the National Health Council. Patients were informed about the research objectives, procedures to be adopted, as well as possible risks and benefits. Individuals were also informed that participation was voluntary and refusal would not cause any penalty or harm regarding their medical or nutrition treatment. All subjects signed an informed consent form.

## RESULTS

**Figure 1:**
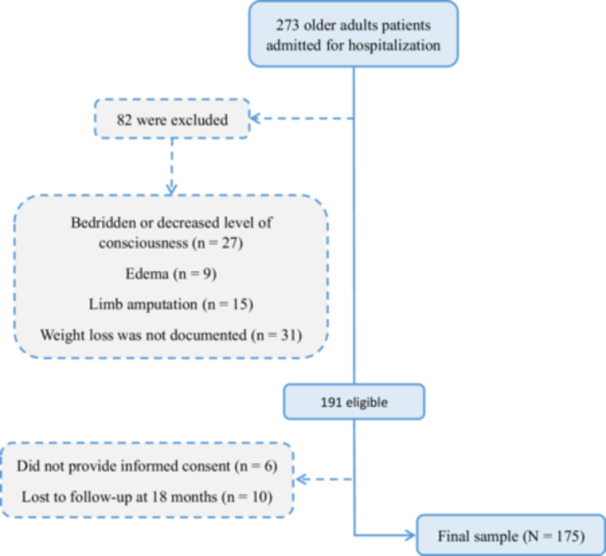
Study flowchart.

In the study period, 273 older adults were hospitalized, of whom 82 were not eligible, and 191 eligible older adults were recruited for the study (Figure 1). However, six of them did not consent to participate and 10 were lost to follow‐up, resulting in a final sample of 175 patients. The average age and sex of the eligible and excluded patients were equivalent (*P* > 0.05).

The median age of the sample was 68 (IQR = 64–75) years, and 56.6% were men. It was observed that 41.7% of the older adults had malignant diseases as the cause of hospitalization, 69.7% had SAH, 41.7% had DM, and 65.7% had some degree of functional dependence.

It was found that 45.7% of the older adults had WS at the baseline of the study, and 46.8% had hospitalization time ≥10 days. In addition, 26.9% of the older adults died, and 43.4% were rehospitalized during the follow‐up.

A higher prevalence of WS was observed in functionally dependent older individuals (PR = 1.3; 95% CI = 1.0–1.7; *P* = 0.040) and in patients who died, with a PR of 1.5 (95% CI = 1.1–2.1; *P* = 0.026). WS was not associated with longer hospitalization (*P* = 0.569) and/or rehospitalizations (*P* = 0.864) (Table 1).

**Table 1:** Distribution of wasting syndrome, according to demographic variables, comorbidities, functionality, and clinical outcomes in older adults patients hospitalized at the Clinics Hospital of the Federal University of Pernambuco, Recife, 2021–2022.

Variables	Wasting syndrome, *n* (%)^a^		PR (95% CI)	*P* value^b^
	No (*n* = 95)	Yes (*n* = 80)		
Sex				0.252
Female	45 (59.2)	31 (40.8)	1.0	
Male	50 (50.5)	49 (49.5)	1.2 (0.9–1.5)	
Age				0.930
60–69 years	54 (54.0)	46 (46.0)	1.0	
≥70 years	41 (54.7)	34 (45.3)	1.0 (0.8–1.3)	
Malignant diseases^a^				0.673
No	54 (52.9)	48 (47.1)	1.0	
Yes	41 (56.2)	32 (43.8)	0.9 (0.7–1.2)	
Hypertension^a^				0.286
No	32 (60.4)	21 (36.9)	1.0	
Yes	63 (51.6)	59 (48.4)	1.3 (0.9–1.5)	
Diabetes mellitus^a^				0.154
No	60 (58.8)	42 (41.2)	1.0	
Yes	35 (47.9)	38 (52.1)	1.3 (0.9–1.6)	
Functionality^a^				0.040
Functional independence	39 (65.0)	21 (35.0)	1.0	
Functional dependency	56 (48.7)	59 (51.3)	1.3 (1.0–1.7)	
Clinical outcomes				
New hospitalization				0.864
No	50 (52.6)	45 (47.4)	1.0	
Yes	41 (53.9)	35 (46.1)	1.0 (0.7–1.3)	
Death				0.026
No	76 (59.4)	52 (40.6)	1.0	
Yes	19 (40.4)	28 (59.6)	1.5 (1.1–2.1)	
Length of hospital stay				0.569
<10 days	50 (55.6)	40 (44.4)	1.0	
≥10 days	42 (51.2)	40 (48.8)	1.1 (0.8–1.4)	

*Note*: *P* < 0.05 is significant.

Abbreviation: PR, prevalence ratio.

^a^ Study baseline. *Malignant diseases*: all types of cancer. *Nonmalignant organic disorders*: digestive disorders, endocrine disorders, infectious diseases, nervous system diseases, respiratory diseases, systemic autoimmune diseases, and kidney and ureteral diseases. *Functionality*: assessed using the Barthel Index. *Wasting syndrome*: unintentional weight loss of ≥5% over a period of 6–12 months.

^b^ Chi‐square test.

**Figure 2:**
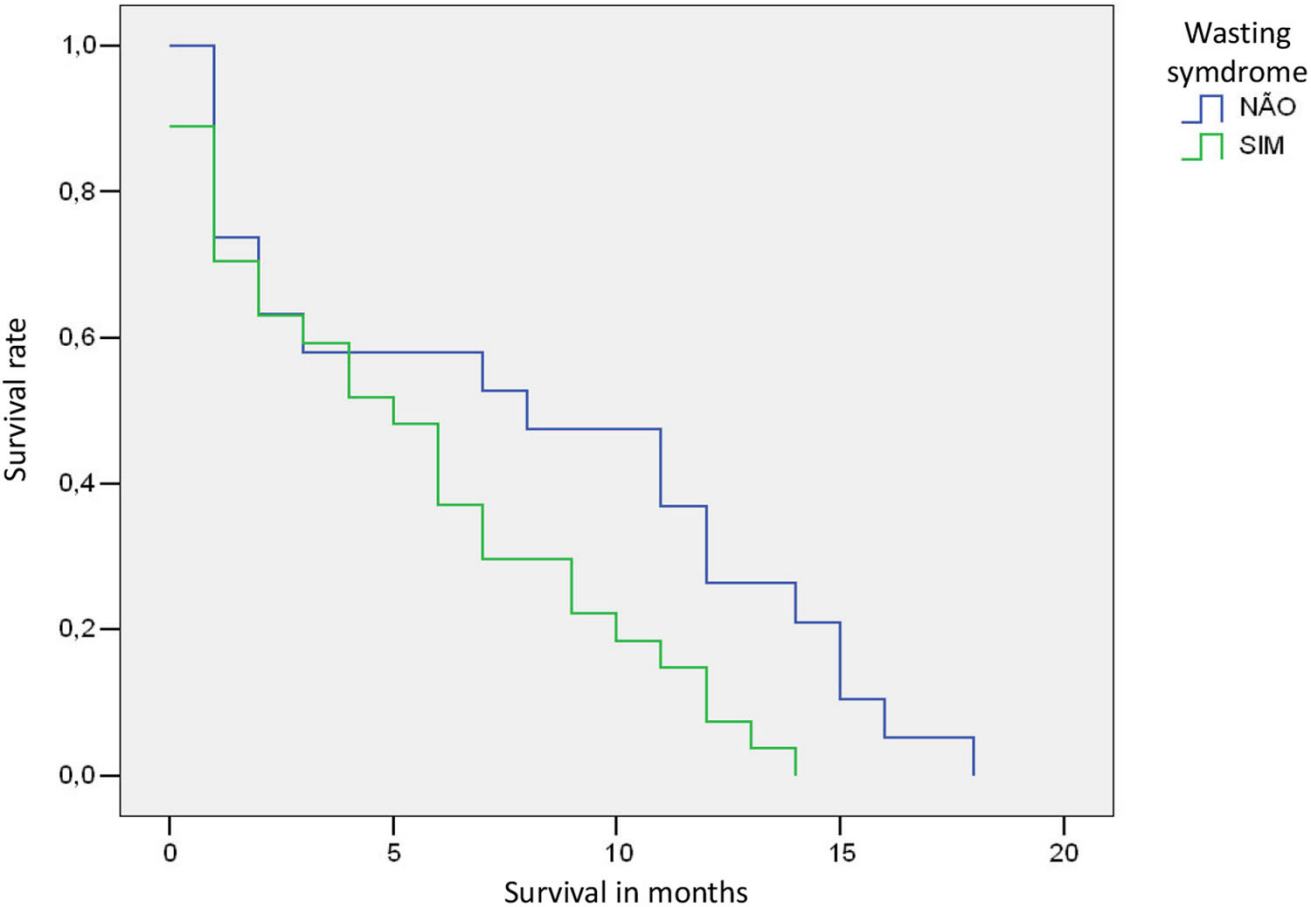
Kaplan‐Meier curves for unadjusted comparisons of mortality by wasting syndrome in older adults from University Hospital, Brazil, 2021–2022. *P value for the log‐rank test (Mantel‐Cox) = 0.028.

The survival analysis, represented by the Kaplan‐Meier curve, indicated that over time deaths occurred earlier among those with WS (*P* = 0.028). It is observed that the differences in the mortality curve behavior between those exposed and not exposed to WS begin to be demonstrated starting from the sixth month of follow‐up (Figure 2).

The assumptions of the Cox model were evaluated, with *P* values > 0.05 for the Cox‐Snell (*P* = 0.876) and Schoenfeld (*P* = 0.258) tests. The proportional hazards model (Cox regression) for the studied population showed that WS lost its effect on mortality when adjusted for age, malignant diseases, and functional dependence (HR = 1.3; 95% CI = 0.7–2.4; *P* = 0.351). Only age remained associated with the outcome, with mortality occurring 2.5 times earlier among older individuals (HR = 2.5; 95% CI = 1.3–4.6; *P* = 0.005) (Table 2).

**Table 2:** Cox regression analysis for 18‐month mortality among hospitalized older adult patients at the Clinics Hospital of the Federal University of Pernambuco, Recife, 2021–2022.

Variable	Mortality			
	Raw HR (95% CI)	*P* value^a^	Adjusted HR (95% CI)	*P* value^a^
Age ≥ 70 years	2.5 (1.4–4.5)	0.003	2.5 (1.3–4.6)	0.005
Malignant diseases	1.4 (0.8–2.5)	0.235	1.4 (0.7–2.4)	0.308
Functional dependency	1.2 (0.6–2.3)	0.511	0.9 (0.5–1.8)	0.821
Wasting syndrome	1.4 (0.7–2.5)	0.298	1.3 (0.7–2.4)	0.351

*Note*: *P* < 0.05 is significant.

Abbreviation: HR, hazard ratio.

^a^ Wald test.

## DISCUSSION

In this study, we present data on WS in hospitalized older patients and its relationship with adverse events up to 18 months. Consistent with previous observations,^13^, ^14^, ^15^ our data showed a clinically significant prevalence of WS (47.5%), and the relationship of this condition with mortality was demonstrated in the survival curves but with the effect lost in the multivariate model. Although most previous studies have primarily focused on demonstrating the relationship between malnutrition and adverse outcomes, this study explored aspects related to involuntary weight loss, a condition that may precede malnutrition and could be the subject of interventions to prevent subsequent nutrition complications.

The high percentage of WS observed in our sample can be partly explained by the fact that malignant diseases, which commonly lead to weight loss, were a very frequent primary cause of hospitalization in our sample, along with high rates of underlying comorbidities and possibly acute medical conditions faced by many patients admitted for hospitalization.

### WS and malignancies

Although some studies suggest cancer or malignancies as possible causes of WS,^2^, ^4^, ^13^, ^14^ this study did not observe a higher frequency of unintentional weight loss in patients with malignant diseases compared with those with nonmalignant diagnoses. This observation may be partly attributed to the fact that these patients were under health monitoring, with treatment in place and had clinically controlled conditions, helping them preserve body weight. Additionally, the stage of the disease and whether the patients were receiving prior nutrition support were not assessed. It is known that oncological diseases require a multidisciplinary approach, and often patients undergo nutrition intervention to control weight loss and recover their condition.

It is important to reflect that weight loss can be a sign of many clinical conditions and may precede the diagnosis of malignancies. Therefore, patients outside the context of malignant diseases in this investigation are not excluded from potential exposure to WS.

### WS and functionality

WS was a predictive factor for functional dependence in older adults, consistent with findings from previous investigations.^15^, ^16^ There are few studies demonstrating this relationship, but one longitudinal study reported that unintentional weight loss ≥5 kg over 1 year was associated with functional decline in ADLs, independently of baseline body mass index, whereas intentional weight loss did not affect the rate of functional change.^16^ In this regard, it is believed that unintentional weight loss often coexists with muscle mass loss, contributing to physical disability and limitation in the development of ADLs among older adults.^17^

### WS and mortality

The findings of the present study revealed that 26.9% of older adults progressed to death within 18 months and that WS may shorten the death outcome, but this effect was attenuated and nonsignificant after adjusting for other predictive variables.

A recent study showed a significant increase in the probability of 30‐day mortality in 110,835 hospitalized patients who experienced unintentional weight loss.^18^ Previously obtained data^19^ also converge with this increased mortality risk associated with WS, as demonstrated in a study of 674 community‐dwelling individuals followed for 6.8 years, in which weight loss was associated with all‐cause mortality.

A plausible explanation for the hypothesis that involuntary weight loss may be related to adverse outcomes, including mortality, is that this condition can be an early sign of various diseases,^18^, ^20^, ^21^ such as cancer, cardiovascular diseases, dementia, Parkinson disease, and other less common causes.^18^, ^21^ It is important to note that unintentional weight loss primarily results in the reduction of muscle and bone mass, and it is already recognized that the amount of skeletal muscle mass plays a crucial role in life expectancy in patients with malignancies. Therefore, this condition may indeed contribute to the increased mortality rate.^22^

In addition, many older adults who experience unintentional weight loss are also prone to malnutrition, which can lead to cachexia, infectious complications, and the development of frailty.^18^, ^22^ All these factors can contribute to adverse outcomes, including an increased risk of premature death.^22^

It is important to consider that unintentional weight loss does not always lead to malnutrition, especially when it occurs in patients with increased body weight. In these cases, this biomarker may be neglected and undertreated, potentially progressing to a state of undernutrition, requiring clinical and healthcare management regardless of nutrition status.

### WS and length of hospitalization and rehospitalization

Little is known about the relationship between length of hospitalization, readmissions, and WS.^23^, ^24^ In the present study, no association was found between this condition and longer hospitalization or occurrence of readmissions. However, the high percentage of prolonged hospitalization (≥10 days) among older adults (46.8%) and the significant rate of readmissions during the study follow‐up (43.4%) highlight the importance of conducting further research with larger samples to assess the impact of WS on these outcomes.

### Future perspectives

As limitations of the study, it should be considered that smoking was not assessed, a potential confounding variable listed in some studies that analyzed the prognostic predictive value of weight loss. Other unassessed variables, such as the presence of clinical symptoms at admission (loss of appetite, fever, and dehydration), concomitant diseases, economic status, alcohol consumption, and physical activity, may also have an effect on the outcome variable and should be considered in future investigations.

Additionally, a reassessment of weight status was not considered during the study follow‐up, making it impossible to identify whether patients with WS remained in this weight loss state or regained the lost weight. Furthermore, it should be considered that the sample was obtained from a single center and in a hospitalization context, limiting the generalization of the results to populations with different characteristics.

It is important to consider that excluding patients with decreased levels of consciousness or physical disabilities such as amputations, paraplegia, tetraplegia, or hemiparesis may interfere with the WS estimates obtained in this study, potentially underestimating the magnitude of the condition observed. Future studies with other assessment strategies, such as bed scales, should be developed, avoiding the exclusion of vulnerable patient groups like those excluded in this study. Additionally, we considered WS based on the difference between the weight measured at hospital admission and the weight reported by the patient, which may introduce recall bias, limiting the accuracy of the information. Future studies should consider medical records, as the information may be more accurate.

On the other hand, it should be emphasized that a longitudinal design was used to establish the relationship between WS and health outcomes, providing important information on how weight status can contribute to the prognostic evaluation of older patients. Few studies have focused on WS in isolation, assessing weight loss in the context of malnutrition, which notably increases the risk of adverse events. Additionally, a sample of older adults who underwent hospitalization was assessed, showing negative outcomes that may follow this period.

## CONCLUSION

This study revealed a high prevalence of WS among older adults hospitalized for admission, affecting nearly half of the sample. This finding indicates the significant magnitude of this health issue in this population. Although WS was not an independent predictor of mortality in the multivariate model, it was associated with earlier deaths in the unadjusted survival analysis. Involuntary weight loss should be addressed in the care plans of older patients, with a focus on prevention and intervention strategies to mitigate its negative effects.

## AUTHOR CONTRIBUTIONS

Roana Carolina Bezerra dos Santos, Cláudia Porto Sabino Pinho, and Alcides da Silva Diniz contributed to the conception and design of the research. Roana Carolina Bezerra dos Santos, Maria Caroline da Silva Patricio, Stefany Beatriz do Nascimento, Letícia Sabino Santos, and Taynara de Sousa Rego Mendes contributed to the acquisition of the data. Cláudia Porto Sabino Pinho and Alcides da Silva Diniz contributed to the analysis and interpretation of the data. Roana Carolina Bezerra dos Santos drafted the manuscript. Alcides da Silva Diniz and Claudia Porto Sabino Pinho critically revised the manuscript. All authors agreed to be fully accountable for ensuring the integrity and accuracy of the work and read and gave final approval for the manuscript.

## CONFLICT OF INTEREST STATEMENT

None declared.
